# Targeting firing rate neuronal homeostasis can prevent seizures

**DOI:** 10.1242/dmm.049703

**Published:** 2022-10-10

**Authors:** Fred Mulroe, Wei-Hsiang Lin, Connie Mackenzie-Gray Scott, Najat Aourz, Yuen Ngan Fan, Graham Coutts, R. Ryley Parrish, Ilse Smolders, Andrew Trevelyan, Robert C. Wykes, Stuart Allan, Sally Freeman, Richard A. Baines

**Affiliations:** ^1^ Division of Neuroscience and Experimental Psychology, School of Biological Sciences, Faculty of Biology, Medicine and Health, University of Manchester, Manchester Academic Health Science Centre, Manchester M13 9PT, UK; ^2^ Institute of Neuroscience, University of Newcastle, Newcastle NE2 4HH, UK; ^3^ Department of Pharmaceutical Chemistry, Drug Analysis and Drug Information, Research Group Experimental Pharmacology, Center for Neurosciences, Vrije Universiteit Brussel, 1050 Brussels, Belgium; ^4^ Geoffrey Jefferson Brain Research Centre, The Manchester Academic Health Science Centre, Northern Care Alliance NHS Foundation Trust, University of Manchester, Manchester, UK; ^5^ Division of Pharmacy & Optometry, School of Health Sciences, Faculty of Biology, Medicine and Health, University of Manchester, Manchester Academic Health Science Centre, Manchester M13 9PT, UK

**Keywords:** *Drosophila*, Seizure, Neuronal homeostasis, Mouse, Pumilio, Translational regulation

## Abstract

Manipulating firing-rate neuronal homeostasis, which enables neurons to regulate their intrinsic excitability, offers an attractive opportunity to prevent seizures. However, to date, no drug-based interventions have been reported that manipulate this type of neuronal homeostatic mechanism. Here, we used a combination of *Drosophila* and mouse, and, in the latter, both a pentylenetetrazole (PTZ)-induced seizure model and an electrically induced seizure model for refractory seizures to evaluate the anticonvulsant efficacy of a novel class of anticonvulsant compounds, based on 4-*tert*-butyl-benzaldehyde (4-TBB). The mode of action included increased expression of the firing rate homeostatic regulator Pumilio (PUM). Knockdown of *pum* expression, in *Drosophila*, blocked anticonvulsive effects of 4-TBB, while analysis of validated PUM targets in mouse brain revealed significant reductions following exposure to this compound. A structure-activity study identified the active parts of the molecule and, further, showed that the pyrazole analogue demonstrates highest efficacy, being active against both PTZ-induced and electrically induced seizures. This study provides a proof of principle that anticonvulsant effects can be achieved through regulation of firing rate neuronal homeostasis and identifies a possible chemical compound for future development.

## Teaser

**Editor's choice:** Epilepsy remains challenging to treat. In this study, we show that manipulating endogenous mechanisms that maintain stability of neuronal activity represents a possible route to better manage this disorder.

## INTRODUCTION

Firing rate neuronal homeostasis provides a possible target to achieve therapeutic control of epilepsy. This is because such neuronal homeostatic mechanisms ostensibly oppose extremes of neuronal activity that are normally associated with seizures. By maintaining neuronal activity patterns at physiologically relevant ‘set points’, neuronal homeostasis maintains stability of both neuron and network function across the life course (Giachello and Baines, 2017). However, to date, neuronal homeostasis, of any kind, has not been specifically targeted for clinical benefit.

Pumilio (Pum) homeostatically maintains action potential firing rates within a set range (Driscoll et al., 2013). Pum is a translational repressor that binds mRNA transcripts and reduces *de novo* protein synthesis, with increased *pum* expression occurring in *Drosophila* neurons exposed to increased synaptic excitation. Conversely, as synaptic excitations fall, *pum* expression is reduced (Mee et al., 2004). The 3′-UTR of Pum-regulated transcripts usually contains one or more copies of a Pum-response element (PRE; UGUANAUA, where N is A, C, G or U) (Gerber et al., 2006). Analysis of both *Drosophila* and mammalian transcriptomes identifies greater than 1000 transcripts that contain one or more PREs, consistent with a broad regulatory role (Zhang et al., 2017). Many of these transcripts are shared across the two animal species, indicative of common regulation by PUM (Bohn et al., 2018; Gerber et al., 2006). However, Pum function as a homeostatic regulator requires additional co-factors (including Nanos and Brain tumor), that may be tissue specific. Thus, the actual effect of PUM, in any species, is likely to be dictated by the presence of co-factors and the number and proximity of additional co-factor-binding elements, in addition to the number of PREs (Arvola et al., 2017; Muraro et al., 2008). In mammals, PUM-regulated transcripts that have potential to influence neuron activity include Na_v_1.6 (*Scn8a*) (Driscoll et al., 2013) and *Glur2* (also known as *Gria2*; AMPA receptor) (Dong et al., 2018). PUM-dependent homeostatic translational repression of Na_v_1.6, in rat cortical pyramidal neurons reduces the amplitude of expressed voltage-gated Na^+^ current (I_Na_) and lowers action potential firing frequency (Driscoll et al., 2013). Downregulation of AMPA receptor expression can also be anticonvulsant, as evidenced by the anti-epileptic compound, perampanel, which is an allosteric antagonist of AMPA receptors (Patsalos, 2015).

Whereas *Drosophila* has one *pum* gene, mammals express two highly similar orthologues (*Pum1* and *Pum2*), which are co-expressed and which bind identical RNA motifs and, thus, appear to act redundantly (Bohn et al., 2018; Galgano et al., 2008; Hogan et al., 2015). Seizure occurrence could reflect reduced homeostatic capability, and it is significant that recent studies suggest that reduced PUM contributes to epilepsy. Specifically, (1) *Pum1* or *Pum2* haploinsufficiency is associated with spontaneous seizures in mice (Follwaczny et al., 2017; Gennarino et al., 2015; Siemen et al., 2011), (2) PUM2 expression is reduced in human patients suffering temporal lobe epilepsy and in rat hippocampus following pilocarpine-induced seizure (Wu et al., 2015), and (3) *pum* expression is reduced in *Drosophila* genetic seizure mutations (Lin et al., 2017). In the latter, transgenic upregulation of *pum* is potently anticonvulsant in these same *Drosophila* mutations (Lin et al., 2017).

Based on a screen to identify chemicals that increase expression and/or stability of Pum, we identified avobenzone, which secondary screens show is anticonvulsant in seizure-sensitive *Drosophila* (Lin et al., 2017). However, the physiochemical properties of avobenzone are not compatible with clinical use. Thus, in this study, we report the identification of an avobenzone analogue, 4-*tert*-butyl-benzaldehyde (4-TBB), that is anticonvulsant and has properties (e.g. solubility) more consistent with clinically active compounds. We show that 4-TBB and analogues [specifically the pyrazole 4-(3,5-dimethyl-1*H*-pyrazol-4-yl)benzoic acid (RAB216)] are active against a range of *Drosophila* seizure mutants and, significantly, reduce severity of both pentylenetetrazol (PTZ)-induced and pharmacoresistant electrically (6 Hz) induced seizures in mouse. Reduction of seizure, in fly and mouse, is accompanied by increased expression of PUM. We further report downregulation of known PUM targets following exposure of mouse brain to 4-TBB, thus validating the proposed mode of action. Thus, this study provides a proof of principle that firing rate neuronal homeostasis can be manipulated for possible anticonvulsant therapy. However, we cannot currently state that 4-TBB (and analogues) only increases PUM expression, which would require full mode-of-action studies.

## RESULTS

### 4-TBB suppresses seizure behaviour in *Drosophila*

**Fig. 1.**
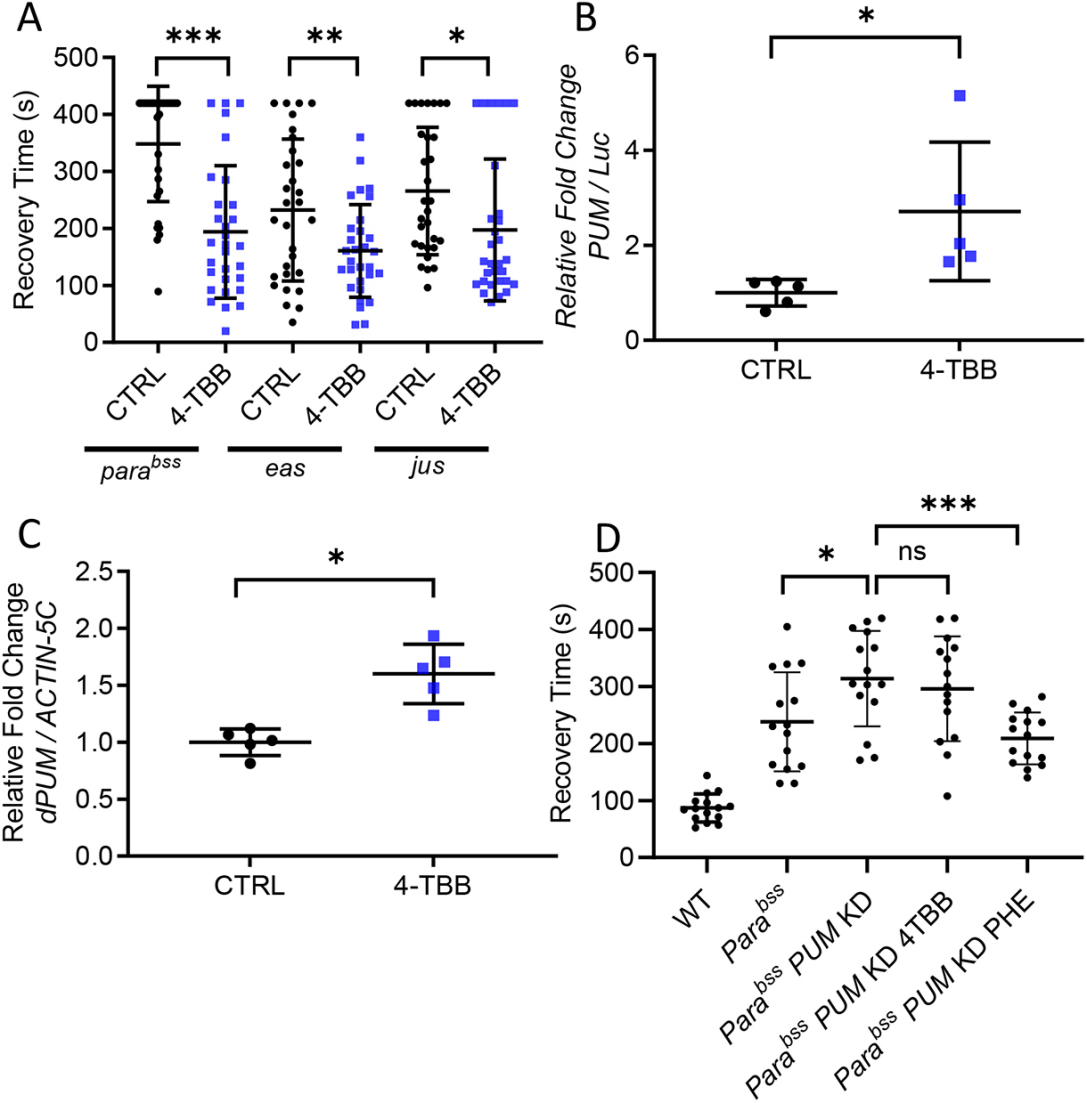
**4-*Tert*-butyl-benzaldehyde (4-TBB) is anticonvulsant in *Drosophila*.** (A) An anticonvulsant effect of 4-TBB is present in three independent *Drosophila* seizure mutations, *para^bss^* (*P*<0.001), *eas* (*P*=0.011) and *jus* (*P*=0.03) (unpaired two-tailed Student's *t*-tests, *n*=30 larvae for each treatment). (B) Identical exposure of transgenic *pum-*promoter-GAL4>UAS-luciferase (Luc) larvae to 4-TBB results in significant upregulation of Luc expression [*P*=0.03, unpaired two-tailed Student's *t*-test, *n*=5 independent samples/10 central nervous systems (CNSs) per sample]. (C) Ingestion of 4-TBB is sufficient to increase *pum* mRNA abundance measured by quantitative RT-PCR (*P*=0.002, unpaired two-tailed Student's *t*-test, *n*=5 independent samples/20 CNSs per sample). (D) RNA interference (RNAi)-mediated knockdown of *pum* expression in *para^bss^* expectedly increases seizure recovery time (Lin et al., 2017) (*P*=0.03). Exposure to 4-TBB is ineffective in this background (*P*=0.87), whereas exposure to phenytoin (PHE; 2 mM in food) remains anticonvulsant (*P*=0.002). Significance tested using a one-way ANOVA [*F*_(4,70)_=24, *P=*0.0009] with correction for multiple comparisons (Dunnett's). Wild-type (WT) recovery time is shown for comparison. Bars show means±s.d. (*n*=15). *P<0.05, **P<0.01, ***P<0.001; ns, not significant. CTRL, control; KD, knockdown.

Single *Drosophila* gene mutations increase seizure-like activity in response to electric shock (Baines et al., 2017). In a prior study, we identified avobenzone to both increase *pum* expression and reduce seizure severity in such *Drosophila* mutations (Lin et al., 2017). Avobenzone is, however, poorly water soluble, and therefore we identified a breakdown product, 4-TBB, to be a better candidate for analysis of mode of action. We tested the anticonvulsant activity of 4-TBB in three diverse seizure mutants to demonstrate wide applicability, regardless of the underlying genetic cause of seizure. Exposure to drug [1.2 mM contained within food, dose available to the central nervous system (CNS) is unknown] was sufficient to reduce electroshock-induced seizure duration in *bang senseless^1^* (*para^bss^*; Na_v_ hypermorph, 194±116 s versus 348±101 s, 4-TBB versus control, *n*=30, *P*<0.001), *easily shocked* (*eas*; ethanolamine kinase deficiency, 160±81 s versus 232±124 s, *n*=30, *P*=0.011) and *julius seizure* (*jus*; encodes a transmembrane domain protein of undetermined function, 197±124 s versus 266±112 s, *n*=30, *P*=0.03; Fig. 1A). The level of seizure suppression observed for 4-TBB, in all mutations, compared favourably to an equal dose of the established anticonvulsant phenytoin (PHE; Fig. 1D), perhaps indicative of equal potency assuming similar pharmacokinetics.

The expression of *pum* is reduced in *para^bss^* mutants, and, moreover, increasing *pum* expression in this mutant background is sufficient to suppress seizure activity in response to electroshock (Lin et al., 2017). To determine whether the anticonvulsant effect of 4-TBB is associated with upregulation of *pum*, we used a *pum*-minimal promoter construct upstream of GAL4 (*pum*-GAL4) to drive expression of UAS-luciferase (UAS-Luc) (Lin and Baines, 2019). Exposure of *pum*-GAL4>UAS-Luc flies to 4-TBB (1.2 mM in fly food) resulted in a significant increase in luciferase activity (2.7±1.5 fold change, *n*=5, *P*=0.03, vehicle control set as 1) (Fig. 1B). We adopted this approach because available anti-Pum antibodies (designed to rodent PUM1 and PUM2) do not work well in *Drosophila*. We also observed a significant increase in *pum* transcript abundance, measured by quantitative RT-PCR (QRT-PCR), of ∼60% (1.6±0.3 fold change, *n*=5, *P*=0.002, vehicle control set as 1) following exposure to the same amount of 4-TBB (Fig. 1C). Finally, we found that the anticonvulsive activity of 4-TBB was significantly diminished when *pum* expression was reduced in the CNS, via targeted expression of an RNA interference (RNAi) construct (Fig. 1D). PHE, which has a different mode of action to 4-TBB (Yaari et al., 1986), remains active under these conditions. We conclude that the mode of action of 4-TBB requires the presence of Pum and increases expression of this homeostatic regulator.

### 4-TBB reduces epileptiform activity in mouse hippocampal culture slices

**Fig. 2.**
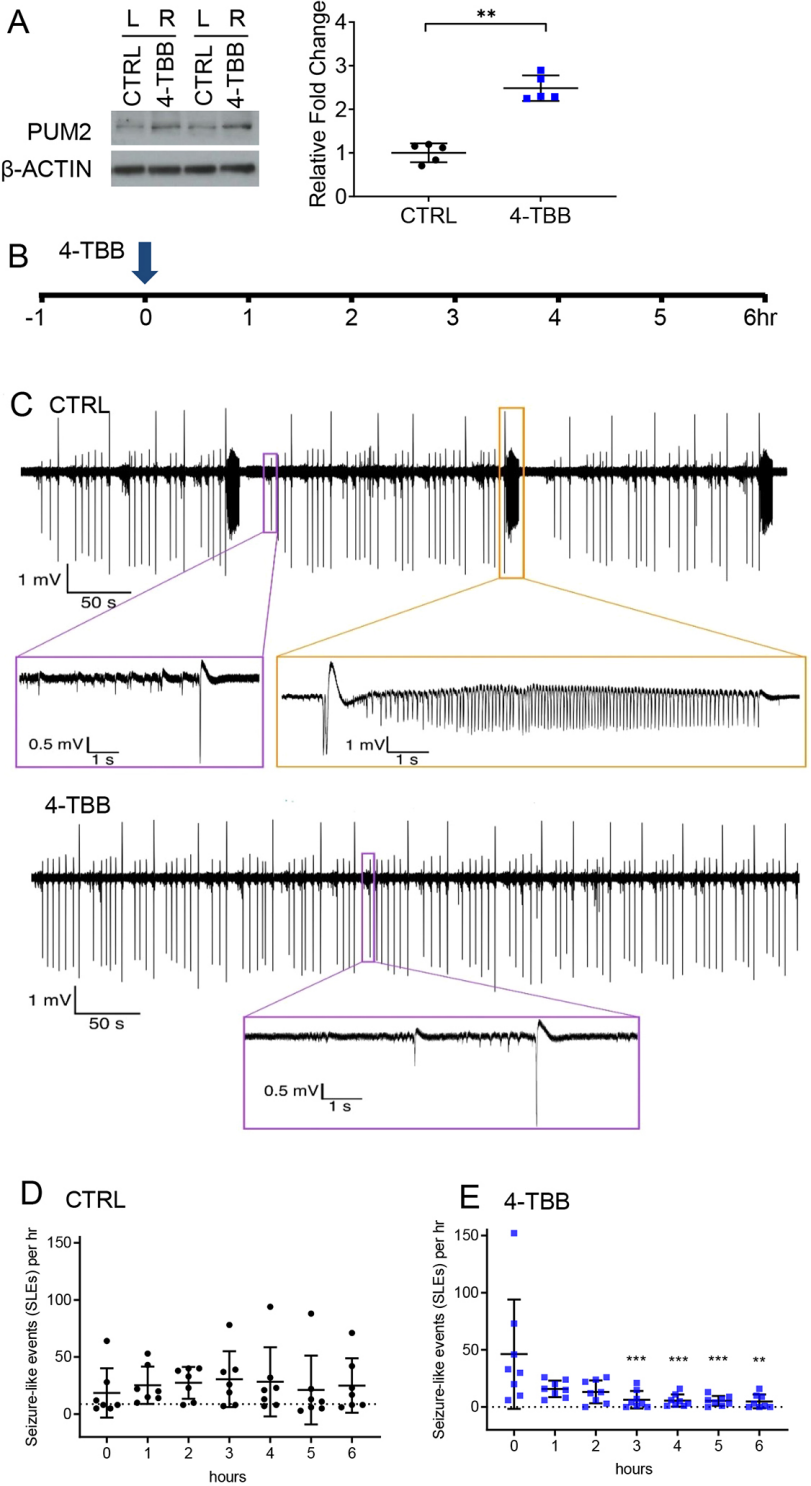
**4-TBB is anticonvulsant in mouse organotypic hippocampal slice cultures.** (A) Incubation (2 h) of mouse brain slice with 4-TBB (1.2 mM) is sufficient to increase expression of PUM2 protein. An example western blot is shown in which the equivalent brain slice, from either hemisphere, was exposed to vehicle (slice from left hemisphere) or drug (slice from right hemisphere). The analysis was repeated five independent times, and these data are shown in the graph (*P*=0.003, paired two-tailed Student's *t*-test). (B) Hippocampal slices [7-14 days *in vitro* (DIV)] that exhibited epileptiform activity were exposed to 4-TBB (1.2 mM in bathing saline) at time ‘0’ during a 7 h recording period. (C) Representative extracellular recordings of local field potentials (LFPs) showing seizure-like activity in the CA1 region of hippocampal slice culture at baseline (CTRL) and 3 h after 4-TBB application (4-TBB). Slice cultures at baseline, and untreated slice cultures throughout the recording period, exhibited short duration interictal spikes (purple boxes) and longer-duration ictal-like events (orange boxes). In this example, 4-TBB treatment eliminated the presence of the longer-duration ictal-like events. (D) An analysis of the number of seizure-like events (SLEs; ictal-like events lasting >5 s) in untreated slices (CTRL) shows robust epileptiform activity throughout the recording period. (E) 4-TBB significantly reduces the frequency of these SLEs [*n*=7 (CTRL) and *n*=8 (4-TBB) independent slices, respectively]. Significance for the effect of 4-TBB *versus* CTRL was tested using a two-way ANOVA [*F*_(13,84)_=5.823, *P<*0.0001]. Analysis of the effect of 4-TBB shows that the reduction in epileptiform activity is significant from 3 h onwards [*F*_(13,91)_=2.473, *P=*0.0063 with correction for multiple comparisons (Šídák's)]. Bars show means±s.d. **P<0.01, ***P<0.001.

Incubation of acutely harvested mouse brain slices with 4-TBB (1.2 mM in the bathing medium) for 2 h was sufficient to produce a significant increase in PUM2 expression (PUM1 not measured), as determined by western blotting (2.5±0.3-fold compared to vehicle-treated controls, which were the corresponding slices taken from the opposite hemisphere incubated in vehicle only, *n*=5, *P*=0.003, Fig. 2A). Addition of a similar amount of 4-TBB (1.2 mM) to mouse organotypic hippocampal slices, in which epileptiform activity was already established (Fig. 2B,C), led to a progressive reduction in epileptiform activity (*P*<0.0001; *n*=7 and *n*=8, control versus 4-TBB, respectively) compared to controls (vehicle only) that was apparent by 1 h and maximal after 3 h (Fig. 2D,E). Following exposure to 4-TBB, a selection of slices was exposed to 4-aminopyridine (4-AP; 100 µm), which, as expected, rapidly induced increased activity (Fig. S1). This demonstrates that slices were healthy following exposure to 4-TBB. We acknowledge that the concentration of 4-TBB used in these experiments was high; however, the sole purpose was to show that this compound could elicit increased PUM expression, and exerts associated anticonvulsive effects, in rodent brain tissue, prior to testing *in vivo*.

### 4-TBB reduces induced PTZ-induced seizure in mice

**Fig. 3.**
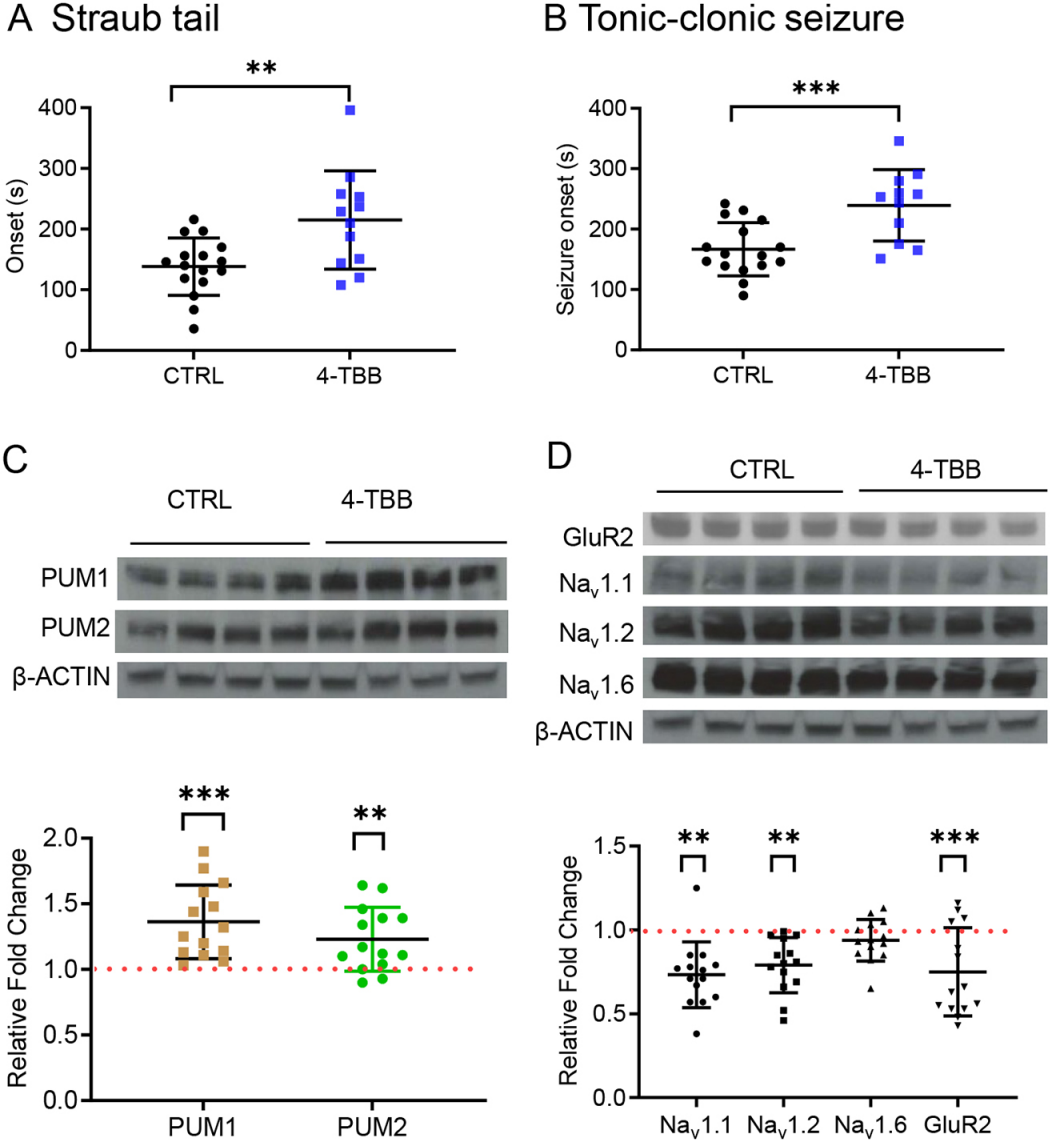
**4-TBB is anticonvulsant in the mouse pentylenetetrazole (PTZ)-induced seizure model.** (A,B) Exposure to 4-TBB increases time to onset of Straub tail (*P*=0.004) (A) and generalised tonic-clonic seizure (*P*=0.001) (B). (C). Post-mortem analysis of brains, taken from the mice used in the assay, shows that expression of PUM1 (*P*<0.0001) and PUM2 (*P*=0.01) is upregulated in animals treated with 4-TBB compared to controls (set as 1). Bars show means±s.d. Inset shows an example western blot. Significance was tested in A-C using unpaired two-tailed Student's *t*-tests. (D) Western blot analysis of Na_v_1.1, Na_v_1.2, Na_v_1.6 and GLUR2 expression in mouse brain shows that exposure to 4-TBB is sufficient to downregulate Na_v_1.1 (*P*=0.005), Na_v_1.2 (*P*=0.007) and GLUR2 (*P*=0.001), but not Na_v_1.6 (*P*=0.75). *n*=14. Bars show means±s.d. Significance was tested using one-way ANOVA [*F*_(4,65)_=6.7, *P=*0.0001] with correction for multiple comparisons (Dunnett's). Inset shows an example western blot. **P<0.01, ***P<0.001.

A standard protocol for PTZ-induced seizures is to apply a test compound immediately (up to 1 h) prior to PTZ exposure (Chen et al., 2011; Erdtmann-Vourliotis et al., 1998). Our preliminary experiments showed that such dosing with 4-TBB [at a maximal dose of 800 mg/kg, subcutaneous (s.c.) injection 1 h prior] was ineffective at preventing PTZ-induced seizures. Thus, to allow longer time for the drug to access the brain, mice were exposed to 4-TBB (800 mg/kg, s.c. injection) or saline vehicle (control), once per day for 3 days prior to PTZ exposure (no other dosing regimens were tested). We observed no overt change in animal behaviour, nor weight loss, during the test period. Four hours after the last injection on day 3, seizures were induced by PTZ (60 mg/kg, s.c. injection). Latency to onset of Straub tailing (a first clear indicator of seizure) was significantly delayed in the 4-TBB-exposed group (215.0±81.0 s versus 138.3±47.3 s, *n*=12 and *n*=16, respectively, *P*=0.004, Fig. 3A). Time to onset of first generalised tonic-clonic seizure (rearing and falling) was also significantly delayed (239±59 s versus 166.8±44.1 s, 4-TBB versus saline control, *n*=12 and *n*=16, respectively, *P*=0.001, Fig. 3B). To confirm the expectation that the anticonvulsive effect of 4-TBB was associated with upregulation of PUM, post-mortem brains were probed by western blotting. Expression of PUM1 and PUM2 was significantly upregulated in 4-TBB-exposed mice (1.4±0.3 and 1.2±0.2 fold increase, *P*<0.0001 and *P=*0.01, respectively, Fig. 3C) compared to the saline controls (set as 1).

Validated PUM-dependent regulated transcripts, in mammals, include Na_v_1.1 (*Scn1a*), Na_v_1.6 (*Scn8a*) and *Glur2* (Dong et al., 2018; Driscoll et al., 2013; Vessey et al., 2010). Bioinformatic analysis of expressed mRNAs also identifies a putative PRE motif in Na_V_1.2 (*Scn2a*), indicative that this channel variant is also regulated by PUM (Bohn et al., 2018). Western blot analysis (Fig. 3D) of the same brain extracts, as above, showed that the expression levels of Na_v_1.1, Na_v_1.2 (0.73±0.2 and 0.79±0.16, *P*=0.005 and *P*=0.007, respectively, *n*=14) and GLUR2 (0.75±0.26, *P=*0.001, *n*=14) were significantly reduced in brain tissue exposed to 4-TBB. No change was observed for Na_v_1.6 (0.94±0.12, *P*=0.75).

### Identification of a more potent 4-TBB analogue

**Fig. 4.**
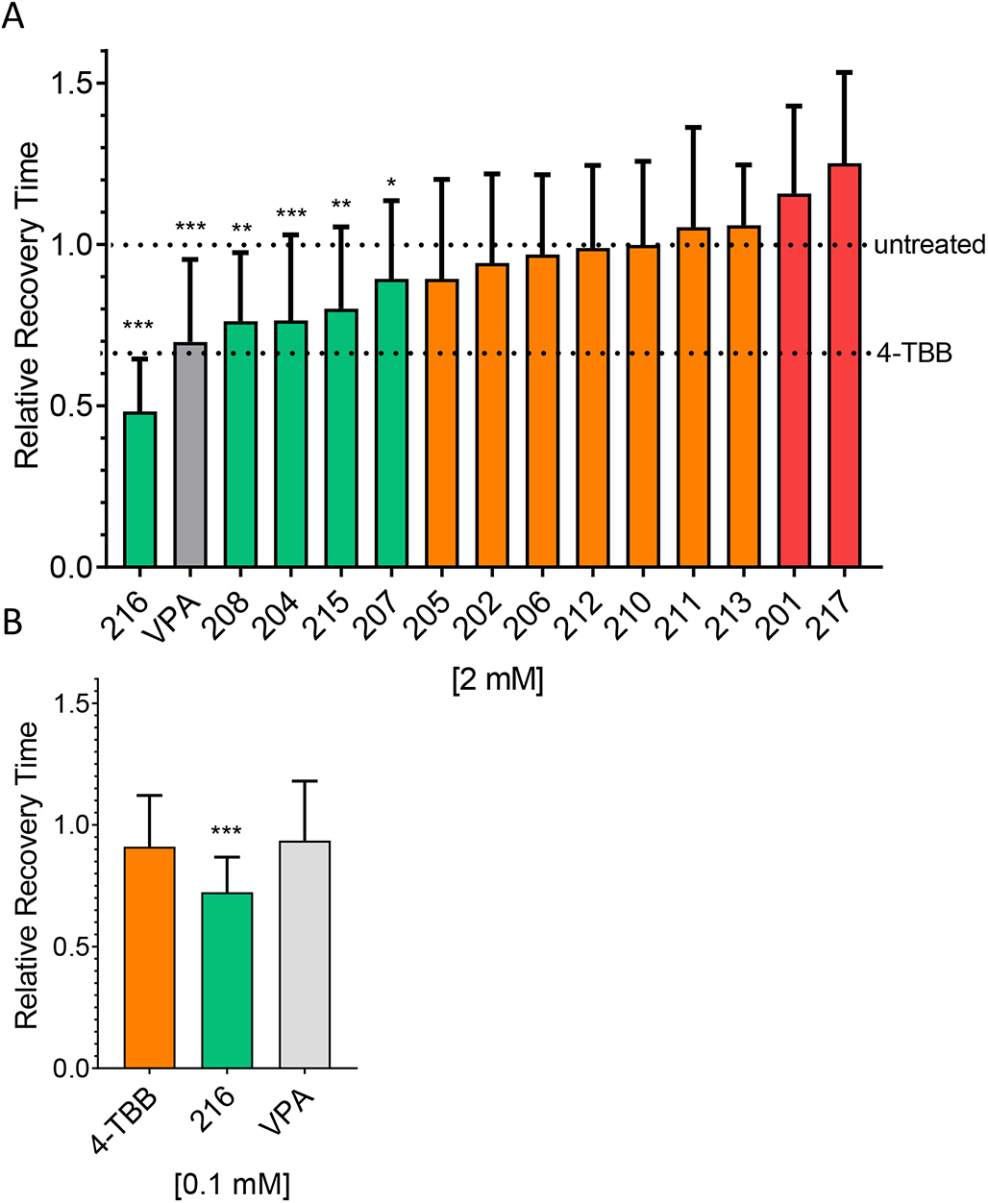
**Identification of a more active 4-TBB analogue.** (A) 4-TBB analogues (structures shown in Table S1) identified a number of active compounds, of which 4-(3,5-dimethyl-1*H*-pyrazol-4-yl) benzoic acid (RAB216) was the most potent. Relative recovery time (recovery time normalised to *para^bss^* run at the same time as drugs) was calculated as a ratio of the treatment group (*para^bss^*+compound) recovery time compared to the corresponding untreated group (*para^bss^* – compound) recovery time from that week of screening. Green denotes a significant reduction in recovery time, orange denotes no change, and red denotes a proconvulsant effect. Sodium valproate (VPA; grey bar) was included as an additional control. *P<0.05, **P<0.01, ***P<0.001 (individual unpaired two-tailed Student's *t*-tests, between *para^bss^* – compound versus *para^bss^*+compound). (B) RAB216 remained active at the reduced concentration of 0.1 mM, whereas both 4-TBB and VPA (tested at the same time) were inactive at this reduced level. Effect of RAB216 was significant at *P*<0.001 compared to 4-TBB. Significance was tested using one-way ANOVA [*F*_(2,89)_=3.9] followed by correction for multiple comparisons (Dunnett's).

The above data show clear proof of principle that a compound able to increase PUM expression has potential to be exploited as an anticonvulsant. However, the active concentration of 4-TBB required for significant effect in the *in vivo* mouse PTZ-induced seizure assay (800 mg/kg) is unacceptably high. Testing 4-TBB at a lower dose (600 mg/kg) reduced seizure behaviour but did not result in statistically significant effects (Fig. S2). To identify a more potent 4-TBB analogue, that may be better suited for clinical use, we exploited *Drosophila para^bss^* to screen for anticonvulsive activity of a diverse set of 14 (synthesised or purchased) 4-TBB-related compounds (Fig. 4A; all structures and chemical properties are shown in Table S1). All compounds were initially tested at 2 mM (concentration in fly food, amount reaching CNS unknown). The commonly used antiepileptic, sodium valproate (VPA; 2 mM), was also included as a positive control. We identified RAB216 to be the most effective analogue against *para^bss^* (Fig. 4A). This analogue was active at 0.1 mM (concentration in fly food), whereas 4-TBB and VPA were inactive at this reduced concentration (Fig. 4B).

**Fig. 5.**
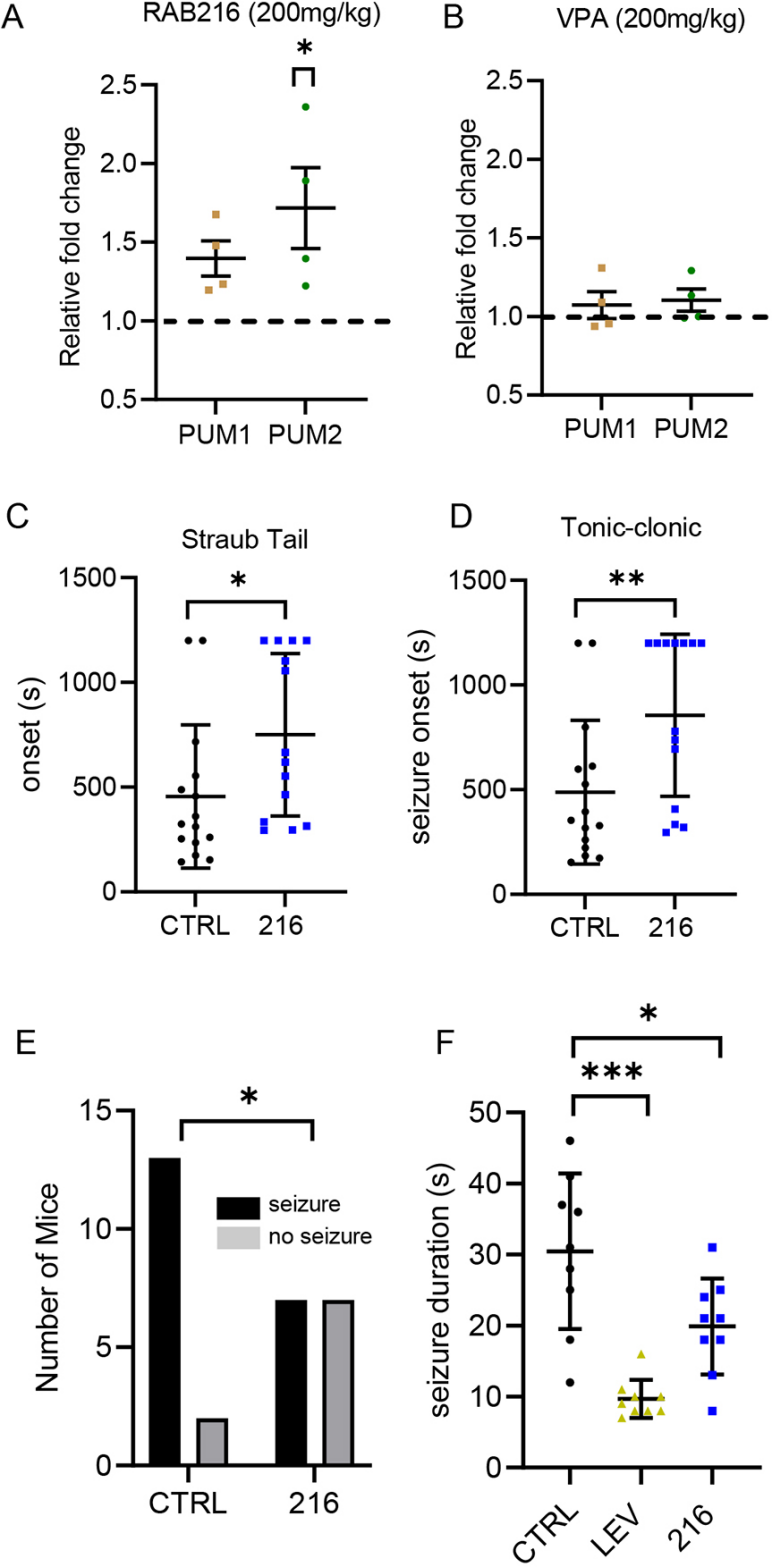
**Characterisation of RAB216 shows it to be more potent than 4-TBB.** (A) Western blot analysis of the effect of exposure of mouse brain to RAB216 on PUM1 (*n*=4, *P*=0.07) and PUM2 (*n*=4, *P*=0.01) expression (unpaired two-tailed Student's *t*-tests). (B) VPA, which is an effective anticonvulsant in this assay, had no effect on expression of PUM1 or PUM2 (*n*=4, *P>*0.05). (C,D) Effect of RAB216 on Straub tail (*P*=0.03) (C) and first appearance of a tonic-clonic seizure (*P*=0.01) in the PTZ-induced seizure assay (D). Timings were capped at 20 min (1200 s). *n*=15 (unpaired two-tailed Student's *t*-tests). (E) Percentage of animals exposed to PTZ showing tonic-clonic seizure following exposure to either saline (CTRL) or RAB216 (*P*=0.05) (*n*=15 and *n*=14, respectively, Fisher’s exact test). (F) Exposure to RAB216 reduces 6 Hz electrically induced seizure duration (*n*=9, *P*=0.013). Levetiracetam (LEV; 100 mg/kg, *n*=9, *P*<0.0001) was used as a positive control with known efficacy in this assay. Significance was tested using one-way ANOVA [*F*_(2,24)_=16.88] with correction for multiple comparisons (Dunnett's). *P<0.05, **P<0.01, ***P<0.001.

Analysis of mouse brain exposed, *in vivo*, to RAB216 (200 mg/kg, once per day for 3 days), revealed a significant increase in PUM2 expression (*P*=0.01) and a smaller (but not significant, *P*=0.07) increase in PUM1 (Fig. 5A). As an additional control for mode of action, we also screened for the effect of VPA (an effective anticonvulsant in the PTZ-induced seizure assay) and saw no change to either PUM1 or PUM2 expression (Fig. 5B). Consistent with its heightened potency to increase PUM2 expression (Fig. 3C), RAB216 was >4× more active than 4-TBB at protecting mice against PTZ-induced seizures, being significantly active at 200 mg/kg (Fig. 5C,D), a dose which prevented the induction of seizure in 50% of animals tested (*P*=0.05, Fig. 5E). By contrast, a repeat of 4-TBB exposure (800 mg/kg), in this assay, prevented tonic-clonic seizures (rearing and falling) in only 30% of animals during the 20 min observation period (not significantly different from control, Fig. S3). This provides confidence that further, yet more active chemical structures, have yet to be discovered, and the results we present here provide a suitable starting point for this ambition.

Finally, we tested RAB216 using a 6 Hz electrically induced seizure assay, which has utility to model drug-refractory epilepsy (Löscher, 2011). Using the same drug administration protocol as for the PTZ-induced seizure assay (i.e. dosing once per day/3 days, then PTZ exposure), RAB216 (200 mg/kg) significantly reduced the length of induced seizure in this assay (*P*=0.013, *n*=9, Fig. 5F). By contrast, 4-TBB (800 mg/kg) did not significantly reduce seizure duration (*P*=0.1, Fig. S4).

## DISCUSSION

We report here that 4-TBB and, particularly, its analogue RAB216 are anticonvulsants with a novel mode of action that involves upregulation of the firing rate homeostatic regulator PUM. Firing rate neuronal homeostasis is likely to assume particular importance in epileptic circuits because of the extreme levels of activity associated with the condition. Notably though, homeostasis has never been specifically targeted for anticonvulsant control. A plausible added appeal for targeting homeostatic mechanisms is that these may be expected to impact rather less on normal physiology. This is because homeostatic mechanisms have multiple in-built protective regulatory controls that work to prevent under- or over-activation; in the case of Pum, this protein also targets its own mRNA and at least one of its cofactors (Muraro et al., 2008) As such, anticonvulsant strategies, involving neuronal homeostasis, may prove less susceptible to side effects, which are the primary reason for switching anti-epileptic medication in the clinic (Kwan et al., 2011). Observation of mice exposed to 4-TBB or RAB216, for 3 days prior to seizure induction, showed no obvious adverse effects. However, we acknowledge that future studies will be needed to screen for possible side effects of the compounds we describe here.

Although multiple forms of neuronal homeostasis have been described, including synaptic scaling and presynaptic regulation of neurotransmitter release (Davis and Bezprozvanny, 2001; Turrigiano and Nelson, 2004), the compounds we describe seemingly manipulate firing rate homeostasis, which acts to maintain action potential firing within pre-determined and physiologically relevant limits (Driscoll et al., 2013). However, the effect may be more complex because it has yet to be established whether neurons utilise multiple forms of homeostasis. If this is indeed the case, then an imposed change to firing rate homeostasis may, in turn, influence synaptic scaling and/or other neuronal homeostatic mechanisms. It has also yet to be established whether all forms of neuronal homeostasis are regulated by separate or overlapping cellular mechanisms. In this regard, it is notable that ketamine and lithium are sufficient to influence synaptic scaling homeostasis (evoking upscaling and downscaling, respectively), perhaps indicative that regulatory mechanisms are separable (Kavalali and Monteggia, 2020). Thus, although the mechanistic details remain to be resolved, our findings suggest that manipulation of homeostasis is a potential therapeutic target to control seizure, most likely by limiting non-physiological levels of neuronal activity.

Firing rate homeostasis is achieved, at least in part, by PUM-dependent control of voltage-gated Na^+^ channel synthesis, in both *Drosophila* and mouse (Driscoll et al., 2013; Mee et al., 2004). Analyses of Na_v_ protein levels, in post-mortem mouse brain pre-exposed to 4-TBB, validates this mode of action, showing reduction in Na_v_1.1 and Na_v_1.2 but, interestingly, no change in Na_v_1.6. Intuitively, one might predict a reduction in Na_v_1.6 because gain-of function mutations in the encoding gene are associated with hyperactivity and epilepsy (O'Brien and Meisler, 2013). By similar logic, the observed reduction of Na_v_1.1 is also unexpected, given that this channel type predominates in GABAergic inhibitory neurons (Catterall et al., 2010). Our analysis of these known PUM targets is, however, relatively crude in treating the whole brain as a single tissue. This approach similarly identifies reduced expression of the GLUR2 AMPA receptor subunit following exposure to 4-TBB. Again, how a reduction in this receptor subunit affects neuronal activity, particularly across the entire brain, is difficult to predict. Glutamatergic synaptic currents, in neurons with reduced expression of GLUR2, exhibit increased deactivation rates, which may limit the degree of depolarisation induced in the postsynaptic cell (Geiger et al., 1995). Although details remain to be resolved (that will require additional experiments), the changes we observe in Na_v_ and GLUR2 protein levels are consistent with increased PUM expression and, in this regard, serve to strengthen our hypothesis that upregulation of this homeostatic regulator contributes to the anticonvulsant effect of 4-TBB and RAB216.

We can, at present, only speculate on how 4-TBB-like molecules mediate an increase in PUM expression. Indeed, in this regard, it is interesting to consider how neurons monitor their activity, which, in turn, is transduced to regulate the activity status of intrinsic homeostatic mechanisms. In the case of Pum, we have reported, in *Drosophila*, that synaptic depolarisation regulates expression of P300 (also known as Nej in *Drosophila*), a histone acetyltransferase that forms a complex with Mef2. As synaptic depolarisation increases, levels of P300 reduce, releasing Mef2 from the complex. Once released, Mef2 binds the *pum* promoter and transactivates gene transcription (Lin and Baines, 2019). In mammals, by contrast, the level of MEF2 expression is itself activity regulated, increasing with depolarisation (Flavell et al., 2006), and analysis of human and mouse *PUM2* promoters identifies multiple MEF2-binding motifs (Lin and Baines, 2019). P300 (also known as EP300 in mammals) is also reported to regulate MEF2 in mammals (De Luca et al., 2003), but how this protein is influenced by synaptic depolarisation has not been described. In mammals, MEF2 also increases the expression of micro-RNAs, including miR-134, which is sufficient to downregulate expression of *Pum2* (Fiore et al., 2009, 2014). Significantly, block of miR-134, using an antagomir, is anticonvulsive in rodents (Reschke et al., 2017). Thus, it is conceivable that 4-TBB, and analogues, might act at any level throughout this seemingly complex regulatory mechanism that ensures appropriate expression of PUM proteins. It is expected that levels of PUM are tightly regulated given the requirement to guard against under- or over-activity of neurons. Indeed, these extensive regulatory and feedback controls, present in PUM-dependent homeostasis, may prove beneficial in exploiting this system for anticonvulsive therapy, minimising potential side effects of exposure to 4-TBB or its analogues.

In summary, this study provides a first proof of principle that manipulation of firing rate neuronal homeostasis provides a possible route to suppress seizures and, moreover, may be suitable for the treatment of patients that have drug-refractory seizures. The expression of PUM is not restricted to the CNS, being instead widespread throughout somatic tissues, and, moreover, PUM is expressed from early development (Goldstrohm et al., 2018). This raises the potential for significant side effects from drugs that act to increase expression of this protein, which may limit the utility of such an approach to control seizures. Thus, targeting of CNS-specific downstream targets of PUM, or CNS-specific PUM co-factors, may prove more successful for clinical control of epilepsy. Clearly, there is much work to be done before the compounds we identify could enter clinical trials, but identification of a potentially druggable neuronal homeostatic mechanism offers the exciting prospect of exploiting a novel target for the eventual control of epilepsy.

## MATERIALS AND METHODS

### Animals

*Drosophila* were maintained at 25°C on a 16:8 h light/dark cycle. Mice were housed on a 12:12 h light/dark cycle at a constant ambient temperature of 21±2°C and given access to water and standard chow *ad libitum*. Mouse PTZ seizure assays used male C57BL/6J mice (12-15 weeks, 23-30 g; Charles River, Margate, UK). For the 6 Hz seizure assay, we used male NMRI mice (10-11 weeks, 35-45 g; Charles River, Écully, France). Drug and vehicle treatment was randomised, and assays were performed by experimenters blinded to treatment. All procedures (as detailed in subsequent paragraphs below) were conducted in accordance with Animal Research: Reporting of *In Vivo* Experiments (ARRIVE) guidelines and local institutional policies and guidelines. All procedures undertaken were approved by the respective University Animal Welfare and Ethics Board (Manchester and Brussels) and conducted in accordance with the respective project licence authority at each institution.

### Seizure behaviour test in *Drosophila*

Wall-climbing, third-instar (L3) larvae, of either sex, were subjected to an electric shock (4 V DC, 3 s) to induce seizure, with or without previous feeding of compound, as described (Marley and Baines, 2011). Recovery times (RTs) are shown in Figs 1 and 4, which depict the time taken for larvae to recover, evidenced by a full peristaltic wave and normal locomotion. A cut-off time of 420 s was used. For compound-feeding studies, eggs were laid on food containing compound [2 mM, or vehicle, 0.4% dimethyl sulfoxide (DMSO)], and larvae were raised (in the presence of drug) until L3. Where experiments were conducted over a number of weeks (e.g. analogue screen shown in Fig. 4), RT was normalised to the *para^bss^* (without compound) run each week.

### *pum* promoter assay

A *pum* promoter-GAL4 line (Lin and Baines, 2019) was crossed to attP24 UAS-Luc flies (Markstein et al., 2008). Flies carrying the UAS-Luc transgene alone were used for background controls. Adult flies were allowed to lay eggs in vials containing food with added compound (or vehicle, DMSO) and to develop to L3. Ten L3 CNSs, of either sex, were placed in 100 µl Promega Glo Lysis buffer for each sample, and five independent samples were collected. CNSs were homogenised, incubated at room temperature (10 min) and centrifuged (3000 ***g***, 5 min), and supernatant was transferred to a new tube. Then, 30 µl of each sample was then transferred to a well of a white-walled 96-well plate at room temperature, 30 µl Promega Luciferase reagent was added to each well, and plates were incubated in the dark (10 min). Luminescence was measured with a GENios plate reader (Tecan, Reading, UK). Values were normalised to total protein concentration, measured using the Bradford protein assay (Bio-Rad, Watford, UK).

### QRT-PCR

QRT-PCR was performed using a SYBR Green I real-time PCR method (LightCycler® 480 SYBR Green I Master, Roche, Mannheim, Germany) as described (Lin et al., 2015). RNA was extracted from 20 L3 CNSs per replicate, of either sex, using an RNeasy micro kit (Qiagen, Hilden, Germany). Primer sequences (5′ to 3′) were as follows: *Actin 5C* (CG4027), 5′-CTTCTACAATGAGCTGCGT-3′ and 5′-GAGAGCACAGCCTGGAT-3′; *pum* (CG9755), 5′-GCAGCAGGGTGCCGAGAATC-3′ and 5′-CGCGGCGACCCGTCAACG-3′ (forward and reverse, respectively). These primers recognise all known splice variants of *pum*. Relative gene expression was calculated as the 2^−ΔCt^, where ΔCt was determined by subtracting the average *Actin 5C* Ct value from that of *pum*. The ratio of target gene expression to housekeeping *Actin 5C* gene (i.e. raw data) was compared across control versus experimental tissue.

### Organotypic slice cultures

Slice cultures were prepared from 5- to 9-day-old C57BL/6J mouse pups, of either sex, according to the interface organotypic culture method (Gogolla et al., 2006; Stoppini et al., 1991). Brains were removed, and hippocampi were dissected and transversely sectioned into 350 µm slices (McIlwain tissue chopper). Slices were plated on polyester membrane inserts (0.4 μm pore) in six-well culture plates (Corning Costar CLS3450-24EA, Sigma-Aldrich, Poole, UK), with two to three slices per insert, containing 1.2 ml of feeding medium (50% minimum essential medium+GlutaMAX, Phenol Red with Earle's salts, Fisher Scientific, Loughborough, UK), 25% heat-inactivated horse serum (Sigma-Aldrich), 21.56% EBSS, 2% B27 serum (Fisher Scientific) and 36 mM D-glucose. Slices were kept at 37°C/5% CO_2_, and the medium was replaced the day after plating and then two to three times weekly depending on plating density. This method of preparing organotypic hippocampal slice cultures induces spontaneous epileptic-like activity without the need for any pharmacological or electrical provocation (Berdichevsky et al., 2012; Ellender et al., 2014; Lillis et al., 2012; Liu et al., 2017). The slicing process mimics a traumatic brain injury, which leads to cell death, deafferentation and subsequent axonal sprouting – all of which contribute towards the gradual development of seizure-like activity (McBain et al., 1989).

Local field potentials (LFPs), at days *in vitro* (DIV) 7-14, were recorded in CA1, in organotypic cultures, using glass borosilicate patch pipettes (∼1-3 MΩ, Harvard Apparatus, Kent, UK) and a Multiclamp 700B (Molecular Devices, San Jose, CA, USA). Slices were perfused with oxygenated ACSF (125 mM NaCl, 26 mM NaHCO_3_, 10 mM glucose, 3.5 mM KCl, 1.26 mM NaH_2_PO_4_, 2 mM CaCl_2_, 1 mM MgCl_2_) and maintained at 33-36°C for 7 h. The first hour was used as an activity baseline: slices lacking seizure-like discharges during this period were discarded. After the first hour of baseline activity, slices were bathed in medium supplemented with 4-TBB (1.2 mM). Analysis was performed using MATLAB (MathWorks, Natick, MA, USA). Signals were digitised using a 1401-3 AD converter (Cambridge Electronic Design, Cambridge, UK), and recorded using Spike2 software (Cambridge Electronic Design, version 7) with sampling rate of 10 kHz. Noise at 50 Hz was removed using an in-built Spike2 software tool, and recordings were discarded if the signal:noise ratio was too great to clearly distinguish events from baseline activity. Interictal events were designated as brief (<1 s), large-amplitude deflections in the LFP trace with a spike and wave discharge pattern. Ictal activity [seizure-like events (SLEs)] followed a similar initial large-amplitude spike and wave discharge followed by a period of after-discharge activity persisting for several to tens of seconds. SLEs were defined as continual discharges lasting >5 s.

### PTZ seizure induction

Mice were injected subcutaneously with 0.1 ml of compound (in NaCl, 0.9% w/v saline) or saline vehicle, once per day for 3 days. Four hours after the last injection on day 3, a single dose of PTZ (60 mg/kg, s.c. in saline) was injected. Each mouse was placed into a separate clear plastic arena and videoed for 20 min. After the observation period, mice were anaesthetised with isoflurane (3-4% in 20% O_2_ and 50% N_2_O, 0.5 l/min) and transcardially perfused with 0.9% saline, and their brains were removed and stored at −80°C. Seizure scoring was carried out from videos, independently scored by two experimenters blinded to the experimental groups until full analysis was complete. Concordance between the two experimenters was high (≤5% difference for timings), and averaged values were taken forward for statistical analysis. Scoring was based on identification of two clear behaviours from a modified Racine score of 3 (Straub tail) and 5 (rearing and falling) (Racine, 1972).

### 6 Hz seizure induction

Prior to the electrical stimulation, 0.5% xylocaine was applied to the cornea to induce local anaesthesia and ensure good conductivity. Corneal stimulation (46 mA, 0.2 ms duration pulses at 6 Hz for 3 s) was administered by a constant current device (ECT Unit 57800, Ugo Basile, Comerio, Italy) (Aourz et al., 2019; Walrave et al., 2018). Acutely evoked 6 Hz seizures were characterised by stun, forelimb clonus, twitching of vibrissae and/or Straub tail. For each animal, the total seizure duration was manually recorded. Intraperitoneal administration of levetiracetam (LEV; 100 mg/kg) 1 h before seizure induction was used as a positive control (Walrave et al., 2015). Seizure scoring was carried out live during the experiment. The entire experiment was also video recorded for confirmation of timings if required. For these experiments, the sole researcher was blinded to the experimental groups until full analysis was complete.

### Western blotting

Whole brain was homogenised in ice-cold buffer (150 mM NaCl, 50 mM Tris-HCl, 1% Nonidet P-40, 0.5% sodium deoxycholate and 0.1% SDS) containing protease inhibitors (Promega, Madison, WI, USA) and centrifuged at 10,000 ***g*** (30 min at 4°C). Supernatant was stored at −20°C. Antibodies were as follows: anti-PUM1 (1:1000, #12322, Cell Signaling Technology, Danvers, MA, USA), anti-PUM2 (1:1000, ab10361, Abcam, Cambridge, UK), anti-SCN1A (1:1000, ASC-001, Alomone Labs, Jerusalem, Israel), anti-SCN2A (1:1000, ASC-002, Alomone Labs), anti-SCN8A (1:1000, ASC-009, Alomone Labs), anti-GLUR2 (1:2000, AB1768-I, Merck, Darmstadt, Germany) and anti-β-actin (1:5000, ab8227, Abcam). Samples (25 µg of protein) were separated by SDS-PAGE, and protein was transferred to a polyvinylidene difluoride membrane (GE Healthcare). After blocking [0.5% bovine serum albumin (BSA) and 0.05% Tween 20 in Tris-buffered saline (TBS-T)], membrane was incubated overnight (4°C) in primary antibody diluted in 0.5% BSA in TBS-T. Membranes were incubated with horseradish peroxidase-conjugated secondary antibody (1:2500, #7074, Cell Signaling Technology) in 0.3% BSA in TBS-T, and blots were developed with an Enhanced Chemiluminescent Detection Kit (Pierce, Rockford, IL, USA). Protein band density was measured using ImageJ (National Institutes of Health, Bethesda, MD, USA).

### Chemical analogues

RAB216 was designed by first screening analogues of 4-TBB and its carboxylic acid derivative RAB102. The structure-activity relationship revealed that only compounds with a carboxylic acid or aldehyde group directly bonded to the benzene ring were active, even when replaced with isosteric groups. Furthermore, a change of the benzene ring to a pyridine or indole ring, or a change of substituents from para to meta, resulted in complete loss of activity. Electronegative groups were tolerated in the para position, increasing the likelihood of a compound forming strong intermolecular bonds with its binding partner. Therefore, when designing the second-generation compounds (Table S1), some analogues included electronegative oxygen and nitrogen atoms. This included RAB216, which contained a pyrazole ring, providing extra interactions to enhance activity. Additionally, analysis of the screen showed that compounds with an element of 3D structure were more active: in RAB216 the two methyl groups attached to the pyrazole group caused it to be twisted by ∼18°. Reviews of drug libraries suggest that planar compounds are less likely to be biologically active (Lovering et al., 2009). Compounds, including RAB216, were designed in accordance with Lipinski's rules, such as molecular mass (<500 Da) and lipophilicity (logP<5), to give desirable physiochemical properties (Lipinski et al., 2001). All of the compounds screened, including RAB216, fit within these guidelines (Table S1).

### Chemical synthesis

#### RAB216

4-Iodobenzoic acid (4.46 g, 20 mmol), copper(I) iodide (0.381 g, 2 mmol), L-proline (0.460 g, 4 mmol) and K_2_CO_3_ (11.10 g, 80 mmol) were suspended in dry DMSO (100 ml) under an argon atmosphere and stirred for 10 min at room temperature. 2,4-Pentanedione (6.2 ml, 60 mmol) was added dropwise to this mixture, which was heated to 90°C and stirred for 24 h. After cooling to room temperature, the mixture was transferred slowly and with stirring into hydrochloric acid (3 M, 250 ml). An additional 150 ml of water was added, and the mixture was cooled in an ice bath, causing a precipitate to form. After filtration and washing with ice-cooled water (3×100 ml), the filter cake was dried in an oven to give 3-(4-carboxyphenyl)-2,4-pentanedione (3.60 g) as a crude product. The compound was taken forward to the next step without purification due to co-elution seen by Thin Layer Chromatography (TLC).

A solution of crude 3-(4-carboxyphenyl)-2,4-pentanedione (3.24 g, 15 mmol) in ethanol (40 ml) was added dropwise to a solution of hydrazine monohydrate (1.75 g, 20 mmol) in ethanol (10 ml) while stirring. Upon complete addition, the combined solution was then heated at reflux for 1 h. After cooling to room temperature, the solvent was removed under reduced pressure to give the crude product. The product was purified by silica gel chromatography (85% EtOAc in hexane) to give RAB216. ^1^H (proton) NMR (nuclear magnetic resonance), 500 MHz (frequency of NMR spectrometer), DMSO-d6 (deuterated solvent) spectrum data: δ (chemical shift) 12.63 (s (singlet), 1H, carboxylic acid), 12.63 (s, 1H, NH), 7.96 (d (doublet), J (coupling constant) = 8.5 Hz, 2H, aromatic protons), 7.42 (d,J = 8.5 Hz, 2H, aromatic protons), 2.24 (s, 6H, 2 methyl groups). ^13^C (carbon) NMR (126 MHz, DMSO-d6) spectrum data: δ 11.6 (2 methyl groups), 127-130, 138.8, 167.5 (carbonyl group). MS (mass spectrometry) [ES^+^ (positive electrospray ionisation), m/z (mass/charge)] 217.1 (molecular ion + H).

#### 4-(1H-Pyrrol-2-yl)benzoic acid (RAB211)

A solution of Na_2_CO_3_ (1.83 g, 17.3 mmol) in water (10 ml) was added to 1-tert-butoxycarbonylpyrrole-2-boronic acid (1.04 g, 4.88 mmol), 4-carboxyiodobenzene (1 g, 4.04 mmol) and tetrakis(triphenylphosphine)palladium(0) (0.30 g, 0.257 mmol) in tetrahydrofuran (THF; 60 ml) under argon. The mixture was heated at 45°C for 2 h and cooled to room temperature. Then, 2 M HCl was added until the solution reached pH 3. The product was extracted with ethyl acetate (3×50 ml) and washed with 0.1 M HCl (3×20 ml). The solution was dried over MgSO_4_, and the solvent was removed *in vacuo* to give 4-[1-(tert-butoxycarbonyl)-1H-pyrrol-2-yl] benzoic acid, which was dissolved in DMSO (20 ml) and heated to 150°C for 30 min. The solution was cooled to room temperature, and 0.1 M HCl (50 ml) was added. The product was extracted with ethyl acetate (3×100 ml) and washed with 0.1 M HCl (3×30 ml). The solution was dried over MgSO_4_ and the solvent was removed *in vacuo*. The product was purified by silica gel chromatography (6% MeOH/CH_2_Cl_2_) to give RAB211 (0.53 g, yield 70%). ^1^H NMR (400 MHz, DMSO-d6) spectrum data: δ 12.76 (s, 1H, carboxylic acid), 11.50 (s, 1H, NH), 7.90 (d, J = 8.7 Hz, 2H, aromatic protons), 7.72 (d, J = 8.7 Hz, 2H, aromatic protons), 6.94 (td (triplet of doublets), J = 2.7, 1.4 Hz, 1H, pyrrole ring), 6.69 (ddd, J = 3.9, 2.5, 1.5 Hz, 1H, pyrrole ring), 6.17 (dt (doublet of triplets), J = 3.5, 2.3 Hz, 1H, pyrrole ring). MS (ES+, m/z) 188.05 (molecular ion + H).

#### 4-(Dimethylcarbamoyl)benzoic acid (RAB212)

Methyl 4-chlorocarbonylbenzoate (1 g, 5 mmol), was dissolved in dichloromethane (DCM; 20 ml), and a solution of dimethyl ammonium chloride (0.61 g, 7.5 mmol) and triethylamine (1.05 ml, 7.5 mmol) was added slowly with stirring. The reaction was left for 1 h and the product was extracted with DCM (3×25 ml) and then washed with water (3×20 ml). The solvent was removed *in vacuo* to give methyl 4-(dimethylcarbamoyl)benzoate as a crude product. The crude product was taken forward to the next step without further purification. Crude methyl 4-(dimethylcarbamoyl) benzoate was dissolved in THF (50 ml) and a solution of LiOH (1 g) in water (50 ml) was added, and the solution was stirred at room temperature for 16 h. HCl was added to the solution with stirring until the solution reached pH 3. The product was extracted with ethyl acetate (3×50 ml) and washed with 0.1 M HCl (3×30 ml) before the solvent was removed *in vacuo* to give RAB212 (0.80 g, yield 83% over two steps). ^1^H NMR (400 MHz, DMSO-d6) spectrum data: δ 7.86 (d, J = 7.7 Hz, 2H, aromatic protons), 7.27 (d, J = 7.7 Hz, 2H, aromatic protons), 2.97 (s, 3H, methyl group), 2.90 (s, 3H, methyl group). MS (ES+, m/z) 194.08 (molecular ion + H).

### Statistics

No data points were excluded from analysis. Following demonstration of normality of raw data, statistical significance was tested using either a Student's two-tailed *t*-test (paired or unpaired), a one-way or two-way ANOVA followed by correction for multiple comparisons, or a Fisher’s exact test (actual test used identified in respective figure legends). For QRT-PCR data, statistical analysis was performed on raw data (i.e. ratio of target gene to *Actin 5C*), prior to normalising for graphical display. *P*≤0.05 was considered significant. Data are shown as means±s.d.

## Supplementary Material

10.1242/dmm.049703_sup1 Supplementary information
