# Supplementary material for: Targeting firing rate neuronal homeostasis can prevent seizures
Source: Dis Model Mech. 2022 Oct 10;15(10):dmm049703. doi: 10.1242/dmm.049703 (PMC9586568; doi:10.1242/dmm.049703)
Supplement: Supplementary information [file dmm-15-049703-s1.pdf]

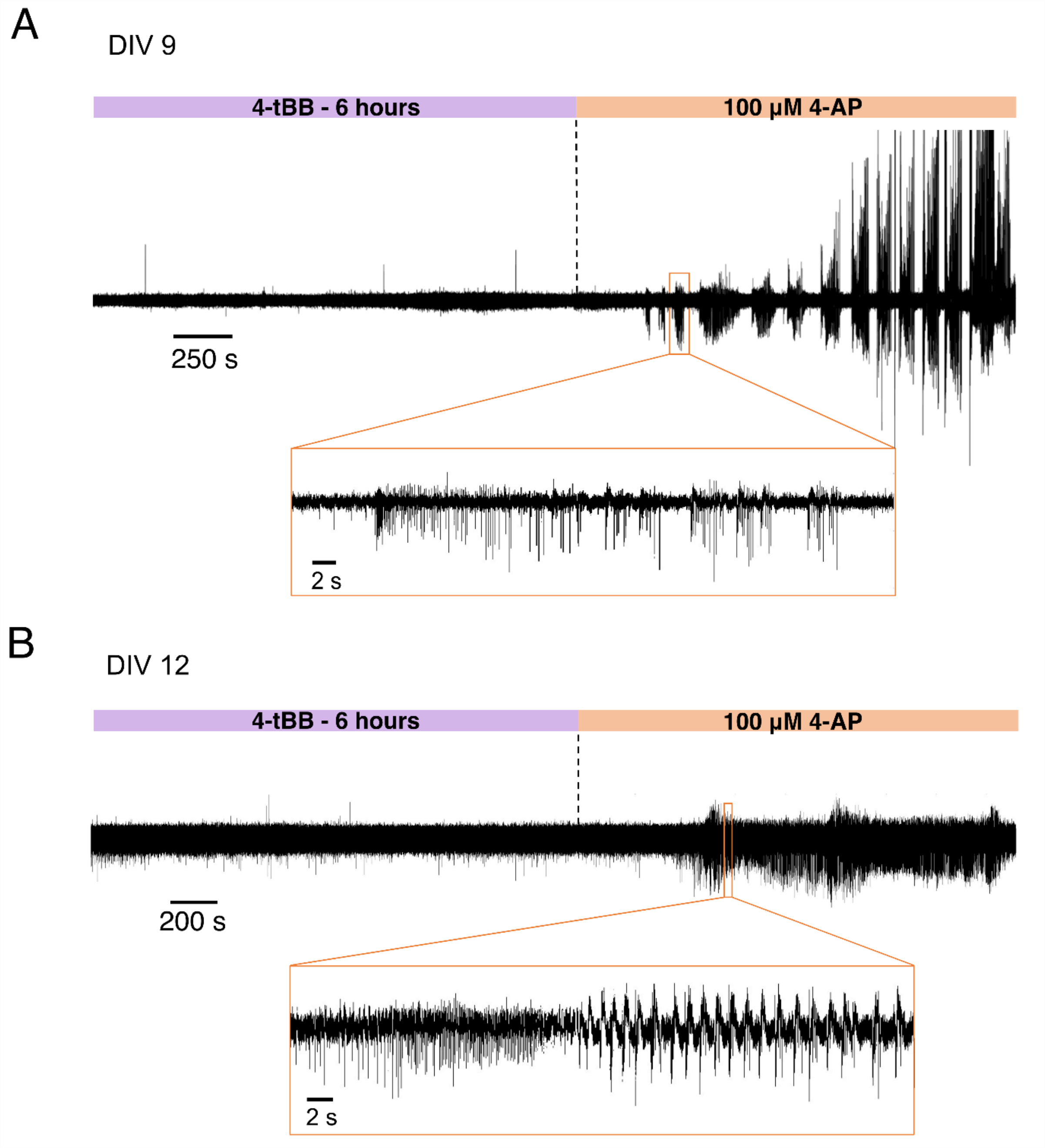

Fig. S1. Application of the K^+ channel blocker 4-aminopyridine (4-AP) shows that slices exposed to 4-TBB are healthy. The addition of 4-AP (100 μM) to slices, following 6hrs exposure to 4-TBB (1.2mM), induces a rapid, and expected, increase in activity. Examples shown are from two independent slices where 4-AP triggered a reappearance of seizure-like activity that had been completely abolished by 4-TBB application.

Disease Models & Mechanisms • Supplementary information

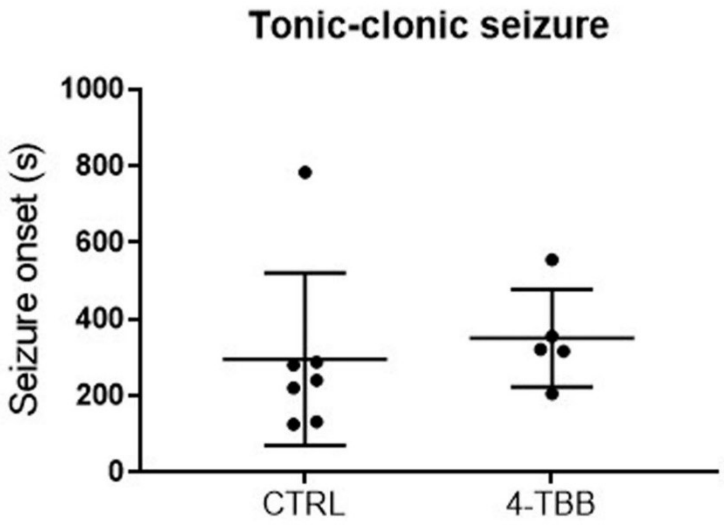

Fig. S2. Effect of 4-TBB exposure on first onset of tonic-clonic seizure.
Exposure to 4-TBB (600 mg/kg) does not significantly reduce time to first onset of tonic-clonic seizure in mice exposed to PTZ (*P* = 0.63, unpaired two-tailed *t*-test).

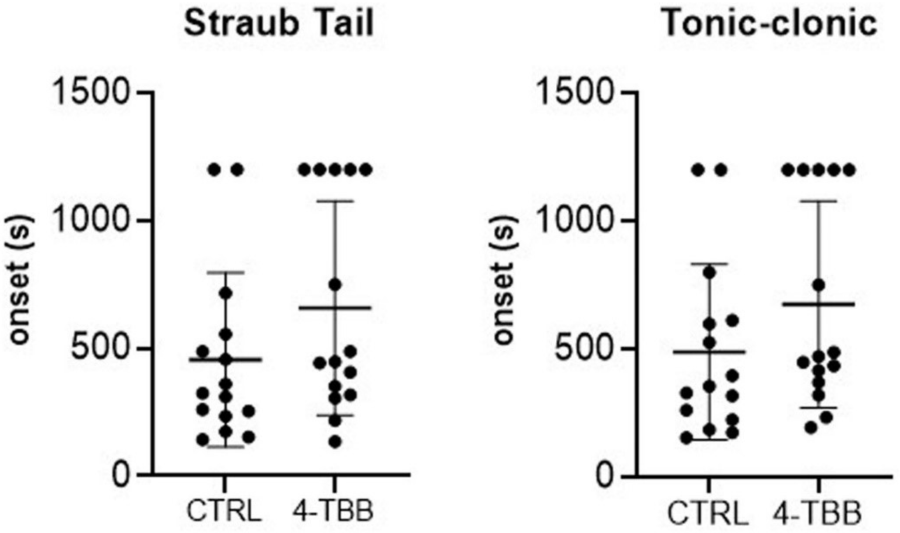

Fig. S3. Repeat of 4-TBB exposure on Straub tail and first onset of tonic-clonic seizure. Effect of 4-TBB (800 mg/kg) on Straub tail (*P* = 0.16, unpaired two-tailed *t*-test) and first onset of tonic-clonic seizure (*P* = 0.18, unpaired two-tailed *t*-test) in the PTZ-induced seizure assay. Timings were capped at 20 min (1200 sec). This amount of 4-TBB prevented tonic clonic seizure in 5 out of 15 mice tested (30%).

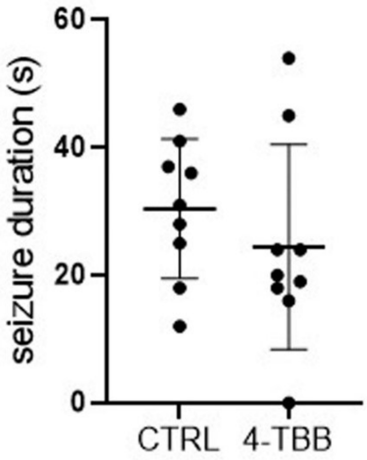

Fig. S4. Effect of 4-TBB exposure on seizure duration. Exposure to 4-TBB (800 mg/kg) does not significantly reduce 6Hz electrically induced seizure duration (*P* = 0.37 unpaired two-tailed *t*-test).

Table S1. Structures and physiochemical properties of 4-TBB analogues screened for anticonvulsive activity in *Drosophila*

Compound	Structure	MW (g/ mol)	CLogP P	Polar surface area Å^2	pKa	Synthesised / Purchased (source + cat no.)
4-TBB	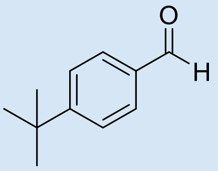	162.23	3.49	17.07	N/A	FluoroChem: 065159
RAB201	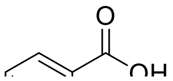	247.99	3.12	37.30	3.8	Merck: ATC311764220
RAB202	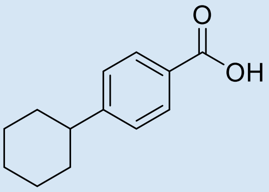	204.11	4.51	37.30	4.3	Merck: AOBH961DC0B4
RAB204	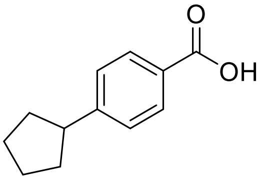	190.09	3.95	37.30	4.3	Merck: AOBH97EBAF06
RAB205	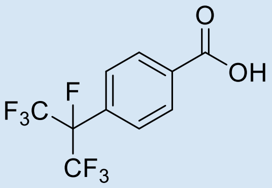	290.01	3.56	37.30	3.0	Merck: ENA514506754
RAB206	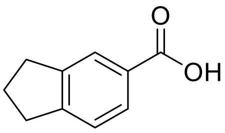	162.06	2.90	37.30	4.1	Fluorochem: 059793
RAB207	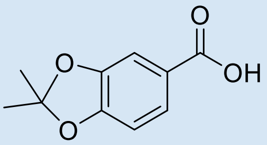	194.05	2.61	55.76	3.9	Merck: COMH04239142
RAB208	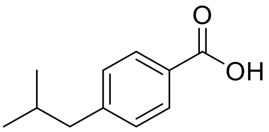	178.09	-3.84	37.30	4.2	Fluorochem: 238558

Disease Models & Mechanisms • Supplementary information

RAB210	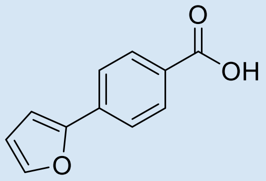	188.04	3.16	46.53	4.3	Fluorochem: 031925
RAB211	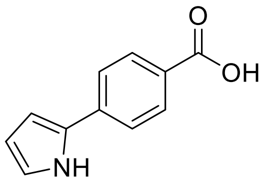	187.06	2.64	49.33	4.4	Synthesised
RAB212	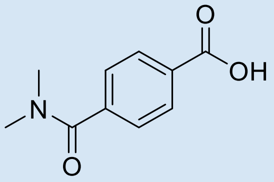	193.07	0.61	57.61	4.2	Synthesised
RAB213	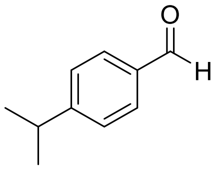	148.08	2.92	17.07	None	Fluorochem: 225406
RAB215	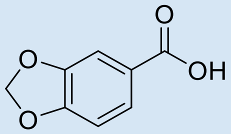	166.02	1.97	55.76	3.8	Fluorochem: 022643
RAB216	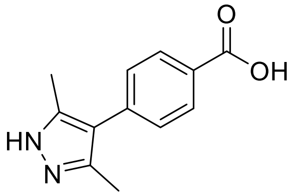	216.24	1.92	61.7	4.0	Synthesised
RAB217	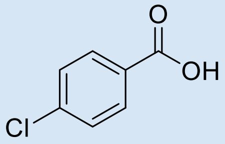	156.57	2.70	37.3	4.0	Fluorochem: 118580

Disease Models & Mechanisms • Supplementary information
